# The Gustatory Signaling Pathway and Bitter Taste Receptors Affect the Development of Obesity and Adipocyte Metabolism in Mice

**DOI:** 10.1371/journal.pone.0145538

**Published:** 2015-12-21

**Authors:** Bert Avau, Dries Bauters, Sandra Steensels, Laurien Vancleef, Jorien Laermans, Jens Lesuisse, Johan Buyse, H. Roger Lijnen, Jan Tack, Inge Depoortere

**Affiliations:** 1 Translational Research Center for Gastrointestinal Disorders, Gut Peptide Research Lab, University of Leuven, Gasthuisberg O&N1, box 701, 3000 Leuven, Belgium; 2 Center for Molecular and Vascular Biology, University of Leuven, Gasthuisberg O&N1, box 911, 3000 Leuven, Belgium; 3 Laboratory of Livestock Physiology, Department of Biosystems, University of Leuven, Kasteelpark Arenberg 30, 3001 Leuven, Belgium; Barnard College, Columbia University, UNITED STATES

## Abstract

Intestinal chemosensory signaling pathways involving the gustatory G-protein, gustducin, and bitter taste receptors (TAS2R) have been implicated in gut hormone release. Alterations in gut hormone profiles may contribute to the success of bariatric surgery. This study investigated the involvement of the gustatory signaling pathway in the development of diet-induced obesity and the therapeutic potential of targeting TAS2Rs to induce body weight loss. α-gustducin-deficient (α-gust^-/-^) mice became less obese than wild type (WT) mice when fed a high-fat diet (HFD). White adipose tissue (WAT) mass was lower in α-gust^-/-^ mice due to increased heat production as a result of increases in brown adipose tissue (BAT) thermogenic activity, involving increased protein expression of uncoupling protein 1. Intra-gastric treatment of obese WT and α-gust^-/-^ mice with the bitter agonists denatonium benzoate (DB) or quinine (Q) during 4 weeks resulted in an α-gustducin-dependent decrease in body weight gain associated with a decrease in food intake (DB), but not involving major changes in gut peptide release. Both WAT and 3T3-F442A pre-adipocytes express TAS2Rs. Treatment of pre-adipocytes with DB or Q decreased differentiation into mature adipocytes. In conclusion, interfering with the gustatory signaling pathway protects against the development of HFD-induced obesity presumably through promoting BAT activity. Intra-gastric bitter treatment inhibits weight gain, possibly by directly affecting adipocyte metabolism.

## Introduction

Obesity is one of the major healthcare problems, affecting millions of people worldwide [1]. Treatment options include life-style changes and pharmacological treatment, but the outcomes are often disappointing and only few drugs can be used on a long-term basis [2]. Therefore, bariatric surgery provides a powerful alternative, resulting in a sustained weight loss and often also remission of comorbidities, such as type 2 diabetes [3]. However, this is an invasive technique, only applied in morbidly obese patients. Thus, there is a need for alternative treatment options.

The gut responds to ingested nutrients through alterations in gastrointestinal motility and the release of gut peptides that help to regulate digestion and absorption but also induce satiation [4]. The drastic changes in body weight observed after bariatric surgery are accompanied by an equally drastic restoration of postprandial gut peptide release, including glucagon-like peptide 1 (GLP-1), peptide YY (PYY) and, although less consistent, ghrelin [5], all dysregulated in obese patients [6, 7]. Therefore, influencing the release of gut peptides might provide a pharmacological alternative to bariatric surgery. The chemosensory pathways involved in the regulation of nutrient-induced gut peptide release have remained elusive for a long time. The discovery of taste receptors and their downstream signaling pathways, including the gustatory G-protein gustducin, on endocrine cells in the gut suggests that they might sense nutrients, much like they do in taste receptor cells on the tongue, to regulate gut peptide release [8, 9]. Indeed, the release of the anorexigenic gut peptides cholecystokinin (CCK) and GLP-1 is regulated by activation of sweet, umami, fatty acid and bitter taste receptors [8–13]. Also the hunger hormone ghrelin is colocalized with the gustatory G-protein subunits, α-gustducin and α-transducin, and with sweet taste and fatty acid taste receptors in the mouse stomach [14–16]. Moreover, intra-gastric administration of bitter agonists induced an α-gustducin-dependent increase in plasma ghrelin levels, accompanied by a short-term increase in food intake [14]. This was however followed by a longer lasting decrease in food intake, correlating with an inhibition of gastric emptying. Vegezzi *et al*. have shown that bitter taste receptors and α-gustducin are differentially expressed in the gastrointestinal tract in response to a high-fat diet in mice [17]. The expression of stomach taste signaling elements has also been reported to be altered in obese patients, with a decreased expression of the sweet/umami taste receptor TAS1R3, but an increased expression of the fatty acid receptor FFAR4 (GPR120), α-gustducin, PLCβ_2_ and TRPM5 [18].

These findings suggest that nutrient sensing via the taste signaling pathway may be altered during nutrient excess. To test this hypothesis, we investigated whether the development of obesity was influenced in mice deficient in the α-subunit of the gustatory G-protein gustducin (α-gust^-/-^) and thus in the taste signaling pathway. Furthermore in view of the inhibitory effect after intra-gastric administration of bitter on food intake in mice [14] and on appetite signaling in humans [19], we investigated whether prolonged intra-gastric administration of bitter agonists influences food intake and hence body weight in high-fat diet induced obese wild type (WT) mice, but not in obese α-gust^-/-^ mice.

## Materials and Methods

### Animals

Wild type C57BL/6 (Janvier) and α-gust^-/-^ (kindly provided by R. Margolskee, Monell Chemical Senses Center, Philadelphia) were kept in the animal facility (2 mice per cage) in a 14–10 light-dark cycle with *ad libitum* access to chow and water. All animal experiments were approved of by and carried out in accordance to the guidelines of Ethical Committee for Laboratory Experimentation (ECD) of the University of Leuven (project: P028/2014). All efforts were made to minimize animal suffering.

### Experimental design

The animals (n = 12–16 per group) were placed after weaning on a high fat diet (60% kcal fat, D12492, Research Diets) for 15 weeks. After becoming diet-induced obese, the animals were treated once daily, 1 hour prior to lights off, by intra-gastric gavage with either denatonium benzoate (60 μmol/kg, Sigma-Aldrich) or quinine-HCl (160 μmol/kg, Fagron) or water as control, during 4 weeks. At the day of sacrifice, the animals were fasted for 6 hours, before receiving a test meal (Nutridrink^®^, Nutricia) by gavage. The animals were anaesthetized with a mixture of xylazine and ketamine, 10 min after receiving the test meal, and sacrificed by cardiac exsanguination and decapitation. Blood samples were supplemented with 1 mM EDTA, 4 mM AEBSF (Sigma-Aldrich) and dipeptidyl peptidase 4 inhibitor (10 μl/ml, Millipore) and were centrifuged. Plasma samples and relevant tissues were collected and stored appropriately for further analysis. The hypothalamus was dissected as a whole using a mouse brain matrix (Zivic instruments).

### Respiratory Quotient and Heat production measurements

Mice were kept in their original cages during the measurements. Gas exchanges were measured in an open circuit indirect calorimetric unit after an adaptation period of 3 days, for 7 consecutive days, as described previously [20]. The respiratory units consisted of three light and temperature controlled climatic chambers, each containing two respiratory cells, a gas analyzer unit and a data acquisition system. The paramagnetic O_2_ analyzer (ADC O2-823A) and infrared CO_2_ analyzer ADC D⁄8U⁄54⁄A) were calibrated before each measurement using gas standards. O_2_ and CO_2_ concentrations from air samples coming out of each cell were measured for 60 s every 15 min over 24 h. The CO_2_ production and O_2_ consumption were calculated from the differences between the gas concentrations of the fresh outside air and the outlet air of each cell. The respiratory quotient was calculated as the ratio of the volume of CO_2_ produced to the volume of O_2_ consumed. Heat production was calculated according to the formula of Romijn and Lokhorst[21]: heat production (kJ/h) = 16.18 O_2_ (l/h) + 5.02 CO_2_ (l/h).

### Quantitative Real-time PCR (qRT-PCR)

Tissues were stabilized in RNA later (Qiagen), total RNA was isolated using the RNeasy mini kit (Qiagen), and treated with the Turbo DNA-free kit (Ambion, Life technologies) to exclude contamination of genomic DNA before reverse transcription using Superscript II Reverse Transcriptase (Invitrogen, Life technologies). The qRT-PCR reaction was performed as described previously, using the Lightcycler 480 (Roche Diagnostics) with the Lightcycler 480 Sybr Green I Master mix (Roche Diagnostics), [22] and analyzed according to the method of Vandesompele *et al*. [23]. Results were expressed relative to the geometric mean of the normalized expression of the three most stable housekeeping genes tested; glyceraldehyde 3-phosphate dehydrogenase (GAPDH), ribosomal protein L13a (RPL13a) and β-actin. The primers used are summarized in S1 Table.

### Radioimmunoassay for octanoyl and total ghrelin

Plasma samples were acidified (10%) with 1 N HCl, immediately after collection. After extraction on a Sep-Pak C18 cartridge (Waters Corporation), samples were dried in a speedvac and the radioimmunoassay was performed as described previously [14].

### Enzyme-linked immunosorbent assay (ELISA) for GLP-1 and Leptin/Insulin

Plasma samples were analyzed for GLP-1 using the active GLP-1 (ver. 2) Kit and for leptin and insulin using the mouse metabolic kit (MesoScale Discovery), according to the manufacturer’s instructions.

### Quantitative adipocyte histology

Adipocyte size was measured on haematoxylin and eosin stained sections of fixated and paraffin embedded gonadal adipose tissue (10 μm) with the Image J open source analysis software. For each tissue, 8 random views on 4 sections were selected and analyzed.

### Western blotting for UCP1

Total protein from WT and α-gust^-/-^ mice adipose tissue was separated using SDS-PAGE and loaded onto a PVDF membrane. The membrane was incubated overnight at 4°C with the primary antibodies: rabbit anti-UCP1 (1:2000, Sigma-Aldrich U6382) and rabbit anti-α-tubulin (1:1000, Thermo Fisher Scientific PA5-22060) as a protein loading control. The secondary antibody used was peroxidase conjugated goat anti-rabbit (1:2000, Dako P0488) (1h, RT). Bands were quantified using relative densitometry and normalized to α-tubulin, using Imagelab 4.0 (Bio-Rad).

### 3T3-F442A differentiation assay

Murine 3T3-F442A pre-adipocytes [24] were grown in basal medium at sub-confluence. Differentiation was induced as described previously [25]. During differentiation, the medium was supplemented with 150 μM DB, 100 μM quinine or vehicle. At regular time points, cell lysates were taken for RNA extraction and the extent of differentiation was quantified from Oil Red O-uptake by lipid containing cells [25]. Data presented are mean of 6–8 observations per condition.

### Statistical analysis

Results are presented as means±SEM. Changes in body weight, food intake, respiratory quotient and heat production over time between different genotypes and treatments were analyzed using a repeated measures mixed models analysis (SAS software package 9). Other data that did not involve multiple measurements over time were analyzed with a one-way ANOVA (3T3-F442A cells) or 2-way ANOVA (mice), followed by planned comparisons post-hoc testing, corrected for multiple testing with the Bonferroni correction (Statistica 12, Statsoft). Significance was accepted at the 5% level.

## Results

### α-gustducin is involved in the induction of obesity during a high-fat diet

**Fig 1:**
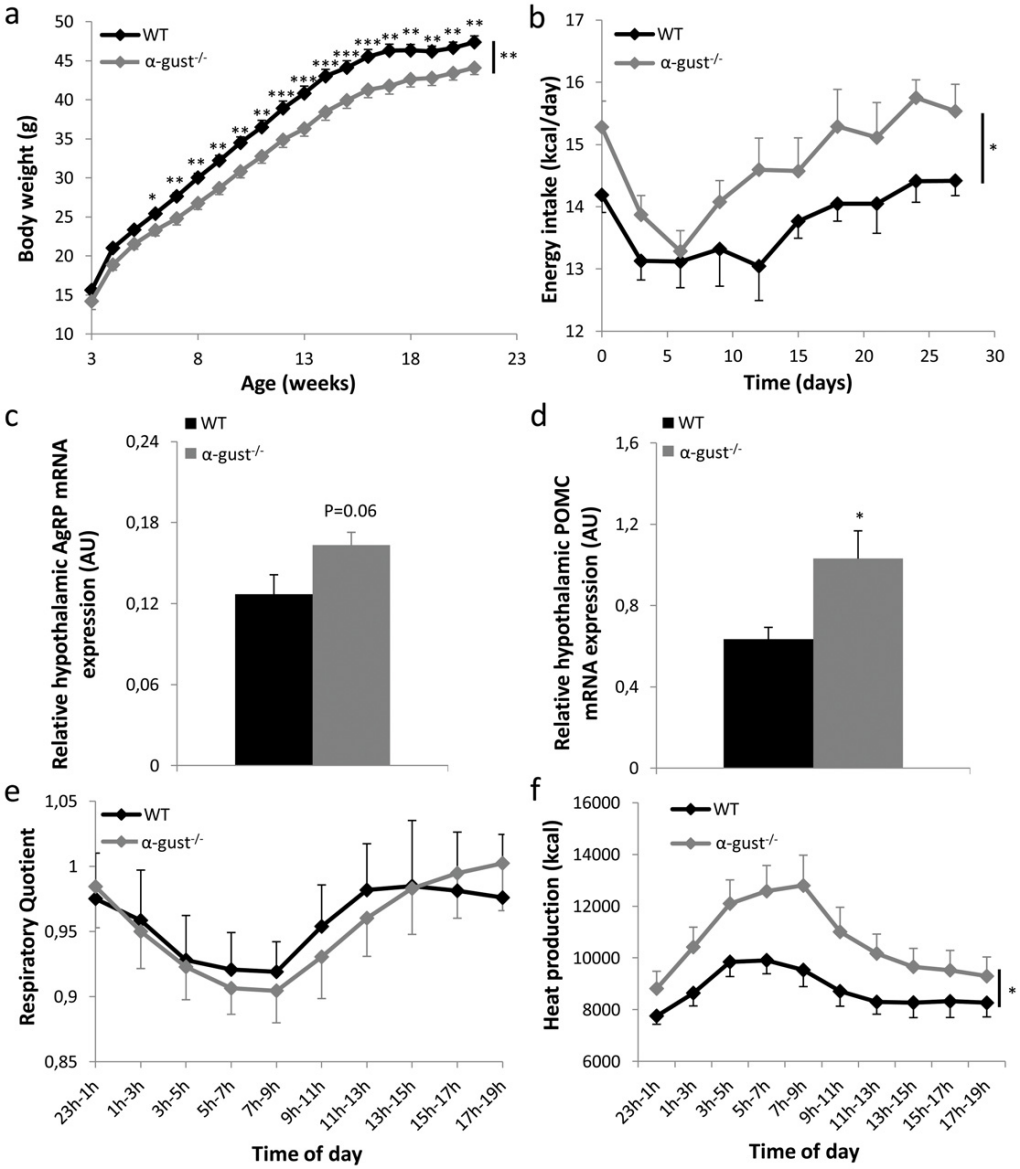
Comparison of the energy balance between WT and α-gust^-/-^ mice on a high fat diet. (a) Time course of body weight of WT (n = 16) and α-gust^-/-^ (n = 16) mice, on a high-fat diet for 19 weeks post-weaning. (b) Energy intake (kcal/day) of WT (n = 16) and α-gust^-/-^ (n = 16) mice during the last 4 weeks before sacrifice. (c-d) Relative hypothalamic AgRP and POMC mRNA levels in WT (n = 10) and α-gust^-/-^ (n = 9) mice after 19 weeks on a HFD. (e-f) Respiratory quotient and heat production, measured continuously during 1 week in WT (n = 8) and α-gust^-/-^ (n = 8) mice. *: P<0.05; **: P<0.01, ***: P<0.001 WT vs α-gust^-/-^.

WT and α-gust^-/-^ mice were put on a high fat diet (HFD) after weaning. Although their initial body weight did not differ, body weight gain started to diverge at week 4 (Fig 1A). Compared to WT mice, body weight of α-gust^-/-^ mice was 9% lower (WT: 49±0.6 g vs α-gust^-/-^: 44.8±0.7 g, P<0.001) after 19 weeks on a HFD. This indicates that the gustatory G-protein subunit α-gustducin is involved in the induction of body weight gain during a HFD.

Energy intake was measured during the last 4 weeks before sacrifice. Despite their lower body weight, α-gust^-/-^ mice consumed significantly more chow than WT mice (P<0.05) (Fig 1B). The mRNA expression of the orexigenic hypothalamic neuropeptide, neuropeptide Y (NPY; P>0.05) was unchanged, while the expression of agouti-related peptide (AgRP; P = 0.06) tended to be higher in the hypothalamus of α-gust^-/-^ mice compared to WT mice (Fig 1C). On the other hand, the hypothalamic mRNA expression of the anorexigenic neuropeptide pro-opiomelanocortin (POMC) was significantly (P<0.05) elevated in α-gust^-/-^ mice (Fig 1D). There were no differences between genotypes in postprandial plasma ghrelin levels (octanoyl ghrelin WT: 15.4±0.8 pg/ml vs α-gust^-/-^:17.5±1.6 pg/ml; total ghrelin WT: 328±10 pg/ml vs α-gust^-/-^: 330±7 pg/ml) and active GLP-1 levels (WT: 0.36±0.1 pg/ml vs α-gust^-/-^:0.44±0.06 pg/ml). Also plasma levels of glucose (WT: 311±8 mg/dl vs α-gust^-/-^ 310±10 mg/dl) and insulin (WT: 2774±299 pg/ml vs α-gust^-/-^: 2427±222 pg/ml) did not differ significantly between genotypes.

After 17 weeks on a HFD, mice were placed for one week in respiratory cells, to measure O_2_ consumption and CO_2_ production. The respiratory quotient did not differ between WT and α-gust^-/-^ mice, indicating no difference in their preference for the substrate used for energy production (Fig 1E). However, the total heat production was significantly higher in α-gust^-/-^ mice compared to WT mice (P<0.05) (Fig 1F).

**Fig 2:**
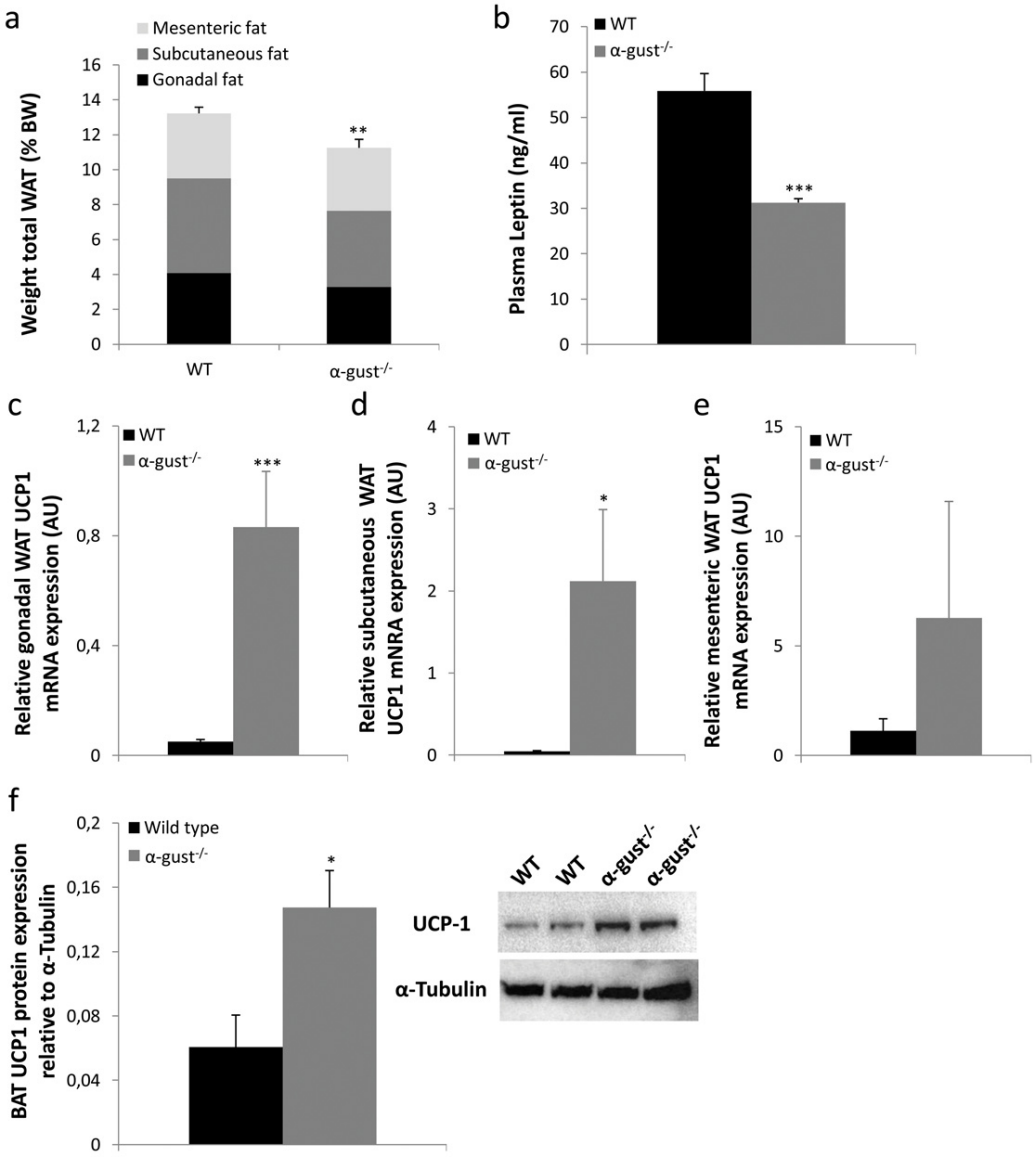
Difference in adiposity between high-fat diet (19 weeks) induced obese WT and α-gust^-/-^ mice. (a) The sum of gonadal, subcutaneous and mesenteric white adipose tissue mass as percentage of total body weight in WT (n = 16) and α-gust^-/-^ mice (n = 16). (b) Plasma leptin levels in WT (n = 10) and α-gust^-/-^ mice (n = 11). (c-e) Relative gonadal, subcutaneous and mesenteric fat UCP1 mRNA levels in WT (n = 6–9) and α-gust^-/-^ mice (n = 6–9). (f) Relative intrascapular brown adipose tissue UCP1 protein level expression in WT (n = 4) and α-gust^-/-^ mice (n = 4) as determined by Western blot. *: P<0.05, **: P<0.01, ***: P<0.001 WT vs α-gust^-/-^.

After 19 weeks on a HFD, mice were sacrificed and different depots of white adipose tissue (WAT) were collected. The sum of the gonadal, subcutaneous and mesenteric fat mass of α-gust^-/-^ mice was 15% lower (P<0.01) compared to that of WT mice (Fig 2A). This difference was mainly driven by differences in gonadal (P = 0.05) and subcutaneous (P<0.05), and to a lesser extent in mesenteric WAT mass (P>0.05). Hematoxylin and eosin stained sections from gonadal WAT were examined histologically. Adipocytes from α-gust^-/-^ mice were significantly smaller compared to adipocytes from WT mice (WT: 6093±71 μm^2^ vs α-gust^-/-^:4913±162 μm^2^; P<0.01). Correspondingly, plasma leptin was significantly lower (P<0.001) in α-gust^-/-^ mice, compared to WT mice (Fig 2B).

The weight of intrascapular brown adipose tissue (BAT) did not differ between genotypes (WT: 290±14 mg, α-gust^-/-^: 253±22 mg). Given the observed difference in heat production, we determined the mRNA expression of uncoupling protein 1 (UCP1), the hallmark of brown adipocytes, in WAT depots of WT and α-gust^-/-^ mice. This was increased in gonadal (P<0.001) and subcutaneous (P = 0.06), but not in mesenteric fat depots of α-gust^-/-^ mice compared to WT mice (Fig 2C–2E). However, at the protein level, this increase in UCP1 expression could not be confirmed in subcutaneous WAT. In addition, also the mRNA expression of other brown adipocyte markers, PR domain containing 16 (PRDM16) and peroxisome proliferator-activated receptor γ coactivator 1 α (PGC1α), was not changed in subcutaneous WAT (S1 Fig). On the other hand, the protein expression of UCP1 was increased 2.4 fold in the intrascapular BAT of α-gust^-/-^ mice (Fig 2F).

### Treatment of obese mice with bitter agonists inhibits body weight gain in an α-gustducin-dependent fashion

**Fig 3:**
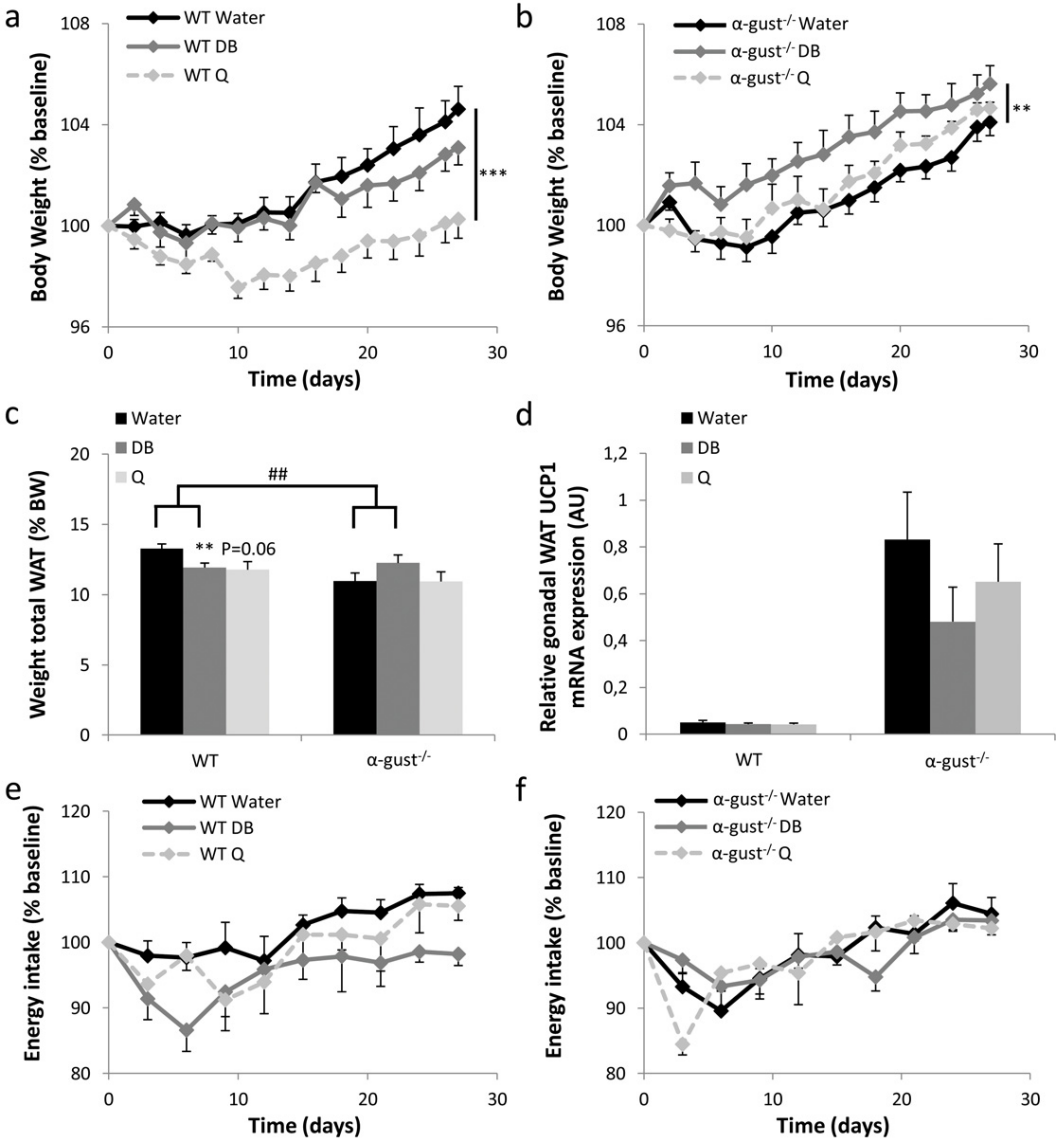
Comparison of the energy balance during treatment with bitter agonists of obese WT and α-gust^-/-^ mice. (a-b) Changes in body weight during daily intra-gastric administration of water, DB (60 μmol/kg) or Q (160 μmol/kg) for 4 weeks in high-fat diet (15 weeks) obese (a) WT (n = 9–12) and (b) α-gust^-/-^ mice (n = 8–12). Results are expressed as percentage change from baseline, defined as the mean body weight measured during one week before the treatment. (c) Combined weight of gonadal, subcutaneous and mesenteric fat pads as percentage of total body weight of control or bitter treated WT (n = 9–12) and α-gust^-/-^ mice (n = 9–12), at sacrifice. (d) Relative mRNA expression of UCP1 in gonadal WAT of control or bitter treated WT (n = 7–9) and α-gust^-/-^ (n = 7–8) mice. (e-f) Changes in energy intake during the 4-week treatment period in (e) WT (n = 6–8) and (f) α-gust^-/-^ mice (n = 7–8), expressed as percentage change from baseline, defined as the mean energy intake measured during 9 days before the treatment. **: P<0.01; ***: P<0.001 water vs bitter; ##: P<0.01 treatment (water vs DB) x genotype.

After 15 weeks on a HFD, mice were gavaged daily for 4 weeks with bitter agonists (denatonium benzoate (DB) or quinine (Q)) or water. WT, but not α-gust^-/-^ mice, treated with DB (treatment x genotype, P<0.01) or quinine (treatment x genotype, P<0.01) lost body weight compared to water-treated mice (Fig 3A and 3B). Quinine was significantly more potent in inducing weight loss in WT mice than DB (P<0.01). In α-gust^-/-^ mice, quinine was without effect but DB even significantly increased body weight (P<0.01). Correspondingly, the combined weight of gonadal, subcutaneous and mesenteric fat was lower in DB (P<0.01) or Q (P = 0.06) treated compared to water-treated WT mice, but not in α-gust^-/-^ mice (Fig 3C, S2 Table). Gonadal WAT was selected for further histological analysis. Both DB and quinine treatment induced a decrease in adipocyte size, with a significant treatment x genotype effect (P<0.05) for DB but not for quinine (S2 Fig). UCP1 mRNA levels in WAT were not changed by treatment with DB or quinine, as illustrated for gonadal WAT in both genotypes (Fig 3D). Bitter treatment did not influence the weight of other organs, such as liver, kidneys, heart and intrascapular brown adipose tissue (S2 Table). In addition, plasma levels of the toxicity markers aspartate transaminase (AST), alanine transaminase (ALT) and alkaline phosphatase were measured, and were not changed, apart from a small increase in ALT in quinine treated WT mice (S3 Fig).

**Fig 4:**
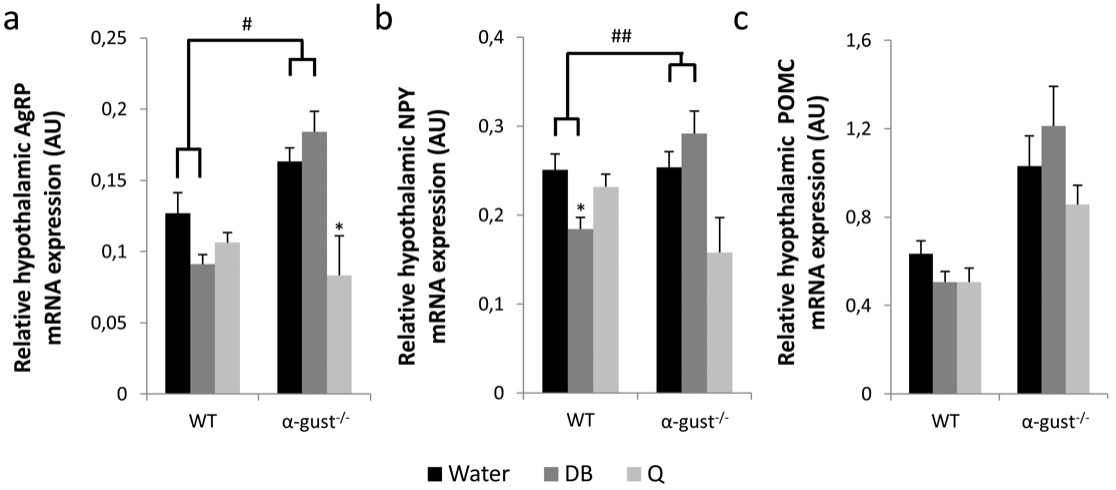
Hypothalamic neuropeptide mRNA expression after treatment with bitter agonists of obese WT and α-gust^-/-^ mice. Relative hypothalamic AgRP (a), NPY (b) and POMC (c) mRNA expression levels in HFD-obese WT (n = 7–10) and α-gust^-/-^ (n = 6–9) mice after 4 weeks of daily gavage with water, DB or quinine. *: P<0.05 water vs DB or Q; #: P<0.05; ##<0.01 treatment (water vs DB) x genotype.

DB-treatment decreased energy intake in WT, but not in α-gust^-/-^ mice, compared to water-treated mice (treatment x genotype, P<0.05) (Fig 3E and 3F). This difference was mainly driven by a decreased energy intake in the first week of the treatment (treatment x genotype, P<0.05). Quinine treatment did not affect energy intake. Correspondingly, the hypothalamic mRNA expression of AgRP (treatment x genotype, P<0.05) and NPY (treatment x genotype, P<0.01) was lower in DB treated WT, but not in DB treated α-gust^-/-^ mice compared to the water-treated animals (Fig 4A and 4B). The mRNA expression of POMC did not differ between treatments (Fig 4C). Quinine had no effect on hypothalamic mRNA expression in WT mice. Bitter treatment had no major influence on respiratory quotient or heat production, during the light as well as during the dark phase (S4 Fig).

### Treatment of obese mice with bitter agonists has minor effects on plasma gut hormone levels

**Fig 5:**
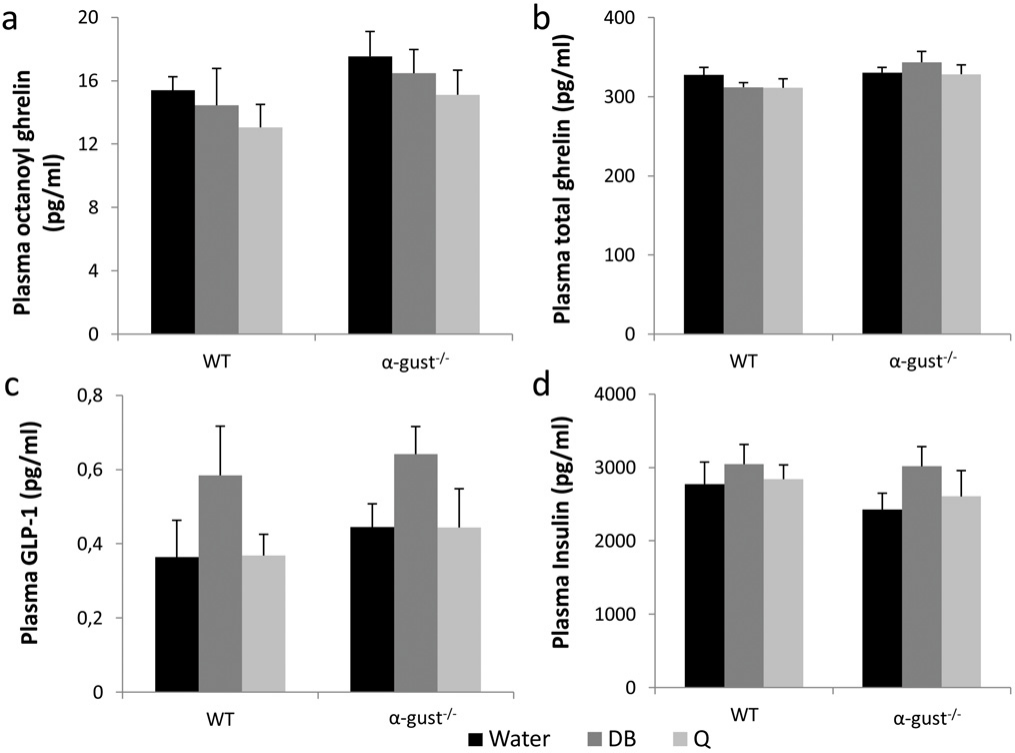
Meal-induced effects on plasma hormone levels after treatment with bitter agonists of obese WT and α-gust^-/-^ mice. (a-b) Plasma octanoylated and total ghrelin levels in control or bitter treated WT (n = 8–11) and α-gust^-/-^ (n = 8–12) mice. (c) plasma active GLP-1 levels in bitter treated WT (n = 9–12) and α-gust^-/-^ mice (n = 10–12). (d) plasma insulin levels in bitter treated WT (n = 6–12) and α-gust^-/-^ (n = 9–12) mice.

Plasma octanoyl and total ghrelin levels measured 15 min after intra-gastric administration of a test meal (Nutridrink^®^) did not differ between treatments or genotypes (Fig 5A and 5B). In contrast, meal-induced plasma levels of active GLP-1 (7-36amide and 7–37) were significantly increased in DB (P<0.05), but not quinine treated animals in a genotype independent manner (Fig 5C). Bitter treatment did not affect plasma insulin or glucose levels in both genotypes (Fig 5D and S5 Fig).

### Bitter agonists inhibit the differentiation of 3T3-F442A pre-adipocytes into mature adipocytes

**Fig 6:**
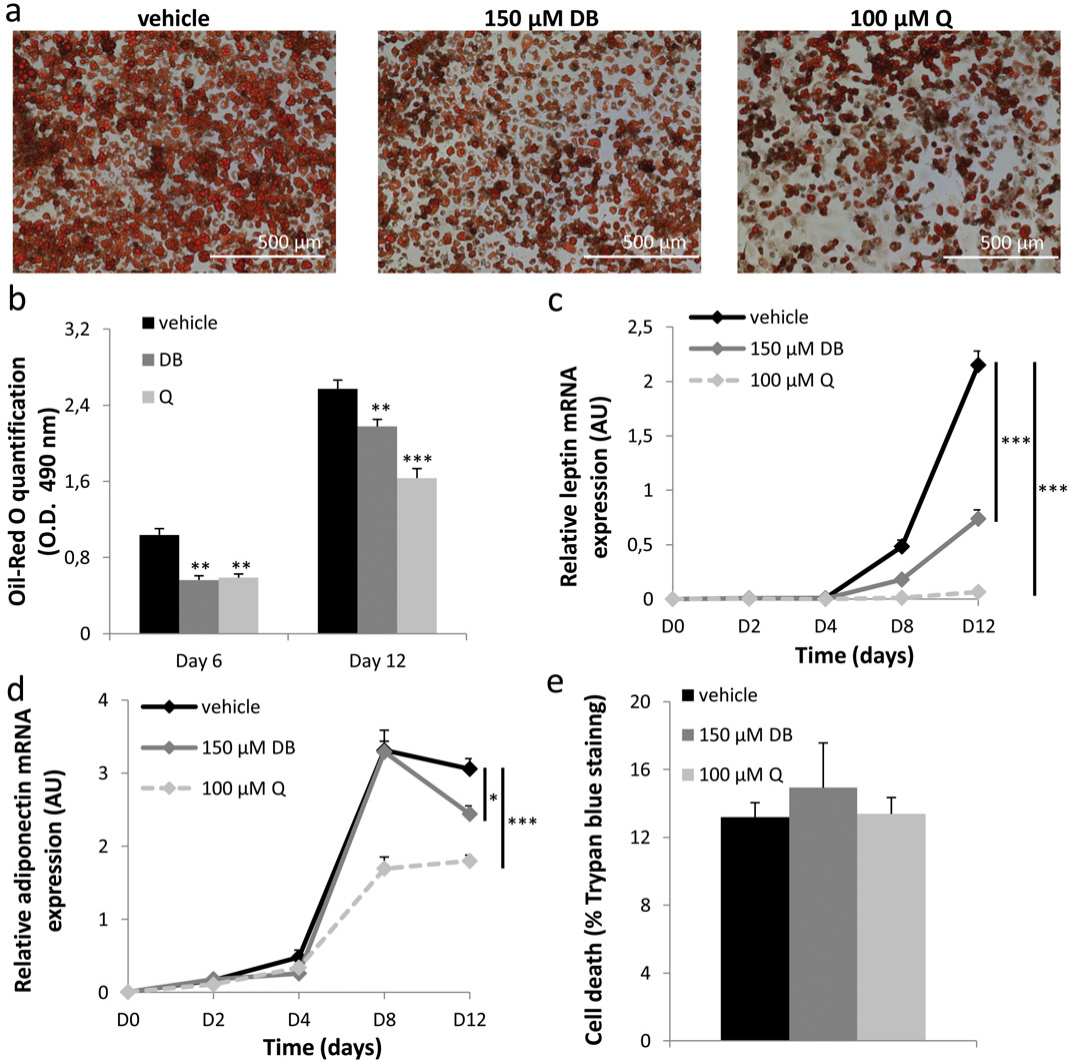
Effect of bitter agonists on differentiation of 3T3-F442A preadipocytes. (a) Representative Oil-Red O stained 3T3-F442A cells at day 12 of differentiation in the presence of vehicle, 150 μM DB or 100 μM Q. (b) Quantification of the Oil-Red O uptake at differentiation day 6 (n = 4) and 12 (n = 8). (c-d) Relative mRNA expression of the markers leptin and adiponectin during differentiation in the absence and presence of DB or Q (n = 6). (e) Trypan blue cell viability assay, presented as the amount of dead cells (% cells stained) after the 12-day differentiation period in the absence and presence of DB or Q (n = 3). **: P<0.01, ***: P<0.001 vehicle vs bitter.

To study a possible direct effect of bitter agonists on adipose tissue, the expression of a selection of bitter taste receptors known to be activated by DB or quinine and their associated G-protein α-gustducin was studied in WAT. mRNA expression for mTas2R108 (DB and quinine), mTas2R135 (DB) and α-gustducin was demonstrated in gonadal, subcutaneous and mesenteric fat of WT mice (S6 Fig). The same TAS2Rs and α-gustducin were also expressed in 3T3-F442A pre-adipocytes (S6 Fig). To study whether bitter agonists could directly affect adipocyte differentiation, 3T3-F442A pre-adipocytes were stimulated to form mature adipocytes in the presence or absence of DB (150 μM) or quinine (100 μM). Cells treated with DB or quinine showed a decreased lipid accumulation, visualized by Oil-Red O staining during differentiation (Fig 6A). DB (P<0.01) and quinine (P<0.01) significantly inhibited lipid accumulation both at day 6, halfway the differentiation protocol, and at day 12 (DB: P<0.01, Q: P<0.001), after full differentiation (Fig 6B). At the gene expression level, mRNA expression for the differentiation markers leptin (Fig 6C), adiponectin (Fig 6D), peroxisome proliferator-activated receptor γ (PPARγ), adipocyte protein 2 (AP-2), fatty acid synthase (FAS) and uncoupling protein 2 (UCP2), but not pre-adipocyte factor-1 (Pref-1) was inhibited after bitter treatment (Table 1). Neither of the treatments was toxic to the cells, as verified by a trypan blue staining (Fig 6E). To test whether the effects of DB and Q were mainly on adipocyte differentiation or rather on metabolism, 3T3-F442A cells were treated either only during the first 4 days of differentiation or alternatively only during the last 8 days with DB or Q. Cells that were incubated only during the first 4 days of differentiation with DB or Q, had no change in lipid uptake at the end of the differentiation period (O.D. at 490 nm: vehicle 0.51±0.01 vs DB 0.52±0.02 vs Q 0.53±0.02, P>0.05). However, if cells were treated from day 4 to day 12 of the differentiation period, a significant reduction in lipid uptake was noted at the end of the differentiation period in response to bitter treatment (O.D. at 490 nm: vehicle 0.48±0.01 vs DB 0.38±0.02 vs Q 0.34±0.03, P< 0.01). These data indicate that bitter agonists directly influence adipocyte metabolism rather than differentiation *in vitro*.

**Table 1:** Relative mRNA expression levels of adipocyte differentiation markers in 3T3-F442A adipocytes after the 12-day differentiation period in the presence of 150 μM DB, 100 μM Q or vehicle.

Differentiation marker	Vehicle (n = 6)	150 μM DB (n = 6)		100 μM Q (n = 6)	
	Relative expression at D12	Relative expression at D12	P-value vs vehicle	Relative expression at D12	P-value vs vehicle
Leptin	2.15±0.13	0.74±0.08	**<0.001**	0.0066±0.0017	**<0.001**
PPARγ	2.51±0.05	1.98±0.07	**<0.01**	2.02±0.06	**<0.001**
AP-2	1.73±0.28	1.44±017	>0.05	1.3±0.09	**<0.01**
FAS	1.07±0.11	0.91±0.07	**<0.05**	0. 8±0.15	**<0.05**
Adiponectin	3.05±0.14	2.44±0.11	**<0.05**	1.80±0.08	**<0.001**
UCP2	1.83±0.1	1.38±0.04	**<0.01**	1.07±0.09	**<0.01**
Pref-1	0.39±0.04	0.38±0.03	>0.05	0.41±0.05	>0.05

## Discussion

The current study investigated the involvement of the gustatory signaling pathway in the development of obesity, by comparing weight gain and food intake in α-gust^-/-^ and WT mice during a high-fat diet. α-gust^-/-^ mice were protected from developing obesity, possibly due to effects on BAT activity, demonstrating the crucial role of α-gustducin in weight gain. Moreover, intra-gastric treatment of these mice with bitter tastants induced weight loss in an α-gustducin-dependent fashion. These results show that interfering with the taste signaling pathway might hold promise for the treatment of obesity.

α-gust^-/-^ mice had a less severe obese phenotype than WT mice after 15 weeks on a high-fat diet as evidenced by their lower body weight, WAT mass and lower plasma levels of leptin. Furthermore, heat production was increased in these mice. Interestingly, gonadal and subcutaneous fat pads of α-gust^-/-^ mice showed an increased mRNA expression of UCP1, the hallmark of brown adipose tissue (BAT) [26]. This suggests the induction of browning of white adipose stores, a concept that has received much attention in recent years as a possible treatment for obesity [27–29]. Unfortunately this observation could not be confirmed at the protein level. However, UCP1 protein levels in the BAT of α-gust^-/-^ mice were increased compared to WT mice. UCP1 is involved in the uncoupling of oxidative phosphorylation from ATP synthesis in the mitochondria of BAT, leading to the generation of heat, which is crucial in the maintenance of body temperature. This result indicates that the taste signaling pathway is involved in the regulation of heat production in BAT. However, the exact location where this regulation occurs remains uncertain.

It is as yet uncertain which taste stimuli are responsible for the differences between WT and α-gust^-/-^ mice, since α-gustducin has been shown to couple to sweet, umami, bitter and even fatty acid receptors [30, 31]. Sweet taste receptors have previously been described on 3T3-L1 pre-adipocytes, and the addition of artificial sweeteners resulted in an inhibited differentiation to mature adipocytes [32]. The authors however found that this was a Gαs-dependent process. In contrast, Simon *et al*. reported a TAS1R-independent increase in adipogenesis in 3T3-L1 cells in response to treatment with artificial sweeteners [33]. Nevertheless, mice deficient in either TAS1R2 or TAS1R3 had a lower body weight compared to WT animals when fed a high-fat high-glucose diet [34]. The animals did, similarly to our experiment, not differ in respiratory quotient, but total heat production was not reported in this study. In addition to possible effects on adipocyte taste receptors, the involvement of other extra-oral taste receptors in for example the gut or central nervous system cannot be excluded. It is also possible that a change in skin thickness or composition might contribute to the observed changes [35]. Examples of genetically modified mice that were shown to have an increased heat production due to an altered skin composition include mice deficient for stearoyl CoA desaturase 1 [36, 37] or fatty acid elongase Elovl3 [38]. In these mice, a clear skin phenotype was reported, including alopecia and a dry skin. α-gust^-/-^ mice on the other hand did not show obvious phenotypic changes in their skin or fur.

Surprisingly, α-gust^-/-^ mice showed higher energy intake than WT mice, despite having a lower body weight. We speculate that this is a compensatory mechanism to adjust for the increased energy expenditure. In contrast, the mRNA expression levels of hypothalamic neuropeptides are inconclusive with the observed difference in energy intake. The mRNA expression levels of the orexigenic neuropeptides AgRP and NPY tended to be higher or were unchanged, respectively, but also the expression of the anorexigenic neuropeptide POMC was upregulated.

Our results clearly indicate that the gustatory G-protein is involved in the induction of obesity. As previous studies have shown that bitter taste receptors can regulate appetite by affecting the release of (an)orexigenic gut hormones and by inhibiting gastric contractility we hypothesized that activation of bitter taste receptors coupled to α-gustducin may represent a strategy to prevent obesity. Mice were treated by oral gavage, to bypass effects of oral TAS2R stimulation and to avoid the induction of aversive responses. Selecting bitter agonists to study *in vivo* effects is challenging, firstly since bitter compounds are often toxic [39, 40], and secondly since there are over 25 different TAS2Rs [41]. DB and quinine were selected because they can activate 8 and 9 different TAS2R subtypes, respectively [42], increasing the likelihood of targeting at least one extra-oral TAS2R. Furthermore, both compounds are known to have a low toxicity [43–46]. Quinine is being absorbed in the blood in mice after intragastric administration [47]. To our knowledge, it is unclear whether denatonium benzoate is being absorbed, however this certainly is the case for benzoate [48].

WT but not α-gust^-/-^ mice treated with DB or quinine showed a decreased body weight gain, indicating that these effects involve the canonical taste-signaling pathway. Changes in postprandial gut peptide levels do not seem to be responsible for the observed changes in body weight after bitter treatment. Meal-induced plasma GLP-1 levels were increased modestly in response to DB but not to quinine in an α-gustducin independent fashion. This indicates that α-gustducin is not the main gustatory G-protein involved in DB-induced GLP-1 release but that also other taste-associated G-proteins such as transducin may be important [49]. Although GLP-1 is an incretin hormone [50], there were no changes in post-meal plasma insulin or glucose levels. One of the limitations of our study is that we could only measure peptide levels at one time point and therefore possible time-dependent changes in meal-induced peptide release cannot be excluded. Thus, although plasma GLP-1 levels peak 15 min after administration of Nutridrink^®^, this time-point is too early to observe a postprandial decline in plasma ghrelin levels [51]. Therefore the ghrelin that was measured rather represents plasma ghrelin levels after a 6h fast which were not affected by long-term bitter treatment. Given that bitter agonists are known to influence gut peptide release *in vivo* acutely, it remains possible that the observed changes on body weight are the result of a summation of acute effects on gut peptide release and changes in meal pattern [11, 13, 14]. In addition, recent studies showed that intragastric administration of DB influences gastric motility both in mice and humans [19]. In humans, the bitter-induced impaired fundic relaxation was associated with increased satiation during an oral nutrient challenge test. Thus it is possible that a change in motility, also involved in appetite generation, might be involved in the overall observed effects [52].

Bitter treatment did not induce major changes in food intake; DB-treatment decreased food intake (mainly during the first week) in an α-gustducin dependent manner, while quinine did not influence food intake at all during the four weeks of treatment. Correspondingly, hypothalamic mRNA expression levels of the orexigenic neuropeptides AgRP and NPY were decreased in DB-, but not water- or quinine-treated WT mice. The fact that gut peptides are not changed drastically is suggestive for a direct effect of DB on the brain, since functional bitter taste receptors for DB have been demonstrated in the brain [53].

Inhibition of body weight gain in lean mice in response to quinine treatment has previously been reported by Cettour-Rose *et al*. [54]. However, in that study, a diet supplemented with quinine was used, thus TAS2Rs in the oral cavity were not omitted. Nevertheless, corresponding to our study, the effects of quinine on body weight were unrelated to changes in food intake. The authors noted a decline in the inhibitory effect of quinine on weight gain in TRPM5^-/-^ mice, a cation channel crucial in taste transduction, further supporting the involvement of taste signaling in the effects of bitter on body weight.

The body weight change observed in our study most likely resulted from a decrease in adipose tissue mass. Therefore, we investigated whether DB and quinine could directly target adipocytes. Our study shows for the first time that TAS2Rs are expressed in white adipose tissue and 3T3-F442A cells. Indeed, the bitter agonists DB and quinine both inhibited 3T3-F442A pre-adipocyte differentiation. Adding bitter compounds during the first 4 days of differentiation did not affect adipocyte differentiation, while addition during the following 8 days did, indicating that the bitter-induced effects are on adipocyte metabolism, rather than on adipocyte differentiation. Future studies need to clarify whether the observed effects *in vitro* are involved in the observations on body weight *in vivo*. Furthermore, it needs to be investigated whether the bitter and sweet taste signaling pathways in adipose tissue might interact with each other to regulate adipocyte function in response to nutrients, much like has been demonstrated on the tongue [55] and in the nasal epithelium [56].

To conclude, chemosensory signaling of nutrients is an important factor in the development of obesity, as mice that have a defective gustatory signaling pathway are less prone to develop obesity. An increased heat production and elevated UCP1 protein indicate an increase in BAT activity in mice lacking α-gustducin resulting in a decrease in WAT mass and hence in lower body weight gain. Furthermore, prolonged treatment of obese mice with DB or quinine induced a reduction in body weight gain and WAT mass that was α-gustducin dependent. Food intake and gut peptide release were not strongly affected, while in vitro studies showed an inhibitory effect of bitter treatment on adipocyte metabolism. The expression of TAS2R mRNA in WAT suggests the possibility of a direct effect of bitter agonists on adipocyte metabolism, which still has to be demonstrated *in vivo*. Therefore, targeting TAS2Rs might show potential to influence adiposity and hence to treat obesity. However, future studies focusing further on the effects of bitter agonists on adipose tissue, using tissue-specific knock-outs, are warranted.

## Supporting Information

S1 Fig Subcutaneous adipose tissue mRNA expression of the brown adipocyte markers PGC1α and PRDM16 in obese WT and α-gust^-/-^ mice. (a) Relative subcutaneous adipose tissue PGC1α (a) and PRDM16 (b) mRNA expression levels in HFD-obese WT (n = 9) and α-gust^-/-^ (n = 9) mice.
(TIF)

S2 Fig Adipocyte size in gonadal adipose tissue after treatment with bitter agonists in obese WT and α-gust^-/-^ mice. Gonadal adipocyte size in HFD-obese WT (n = 5) and α-gust^-/-^ (n = 6) mice. *: P<0.05 water vs DB; $ $: P<0.01 WT vs α-gust^-/-^; #: P<0.05 treatment (water vs DB) x genotype.
(TIF)

S3 Fig Plasma levels of the toxicity markers aspartate transaminase (AST), alanine transaminase (ALT) and alkaline phosphatase after treatment with bitter agonists in obese WT and α-gust^-/-^ mice. Plasma levels of AST (a), ALT (b) and alkaline phosphatase (c) in HFD-obese WT (n = 8–14) and α-gust^-/-^ (n = 9–13) mice. *: P<0.05 water vs Q.
(TIF)

S4 Fig Respiratory quotient and heat production during bitter treatment in obese WT and α-gust^-/-^ mice. (a-b) Mean respiratory quotient and (c-d) heat production (area under the curve) measured continuously during 1 week in (a, c) the dark and (b,d) the light phase in ad-libitum fed WT (n = 8) and α-gust^-/-^ (n = 8) mice, treated with water, DB or Q for 4 weeks. *: P<0.05 water vs DB.
(TIF)

S5 Fig Meal-induced effects on plasma glucose levels after treatment with bitter agonists in obese WT and α-gust^-/-^ mice. Plasma glucose levels in bitter treated WT (n = 9–12) and α-gust^-/-^ (n = 8–10) mice.
(TIF)

S6 Fig RT-PCR transcripts coding for taste signaling molecules in mouse adipose tissue and 3T3-F442A cells. (a) RT-PCR transcripts coding for mTAS2R108 (DB and Q), mTas2R135 (DB) and α-gustducin in obese WT mouse gonadal, subcutaneous and mesenteric fat pads. (b) RT-PCR transcripts coding for mTas2R108, mTas2R135 and α-gustducin in 3T3-F442A cells. Samples in which no reverse transcriptase was added upon production of the cDNA were used as negative control (RT-).
(TIF)

S1 Table Primer sequences used for qRT-PCR. (DOCX)

S2 Table Average tissue weights of obese WT and α-gust^-/-^ mice, treated intra-gastrically with water, DB or Q for 4 weeks. *: P<0.05 water vs Q; $ $ $ P<0.001 WT vs α-gust^-/-^.
(DOCX)
