# Supplementary figures and images for: The Gustatory Signaling Pathway and Bitter Taste Receptors Affect the Development of Obesity and Adipocyte Metabolism in Mice
Source: PLoS One. 2015 Dec 21;10(12):e0145538. doi: 10.1371/journal.pone.0145538 (PMC4686985; doi:10.1371/journal.pone.0145538)

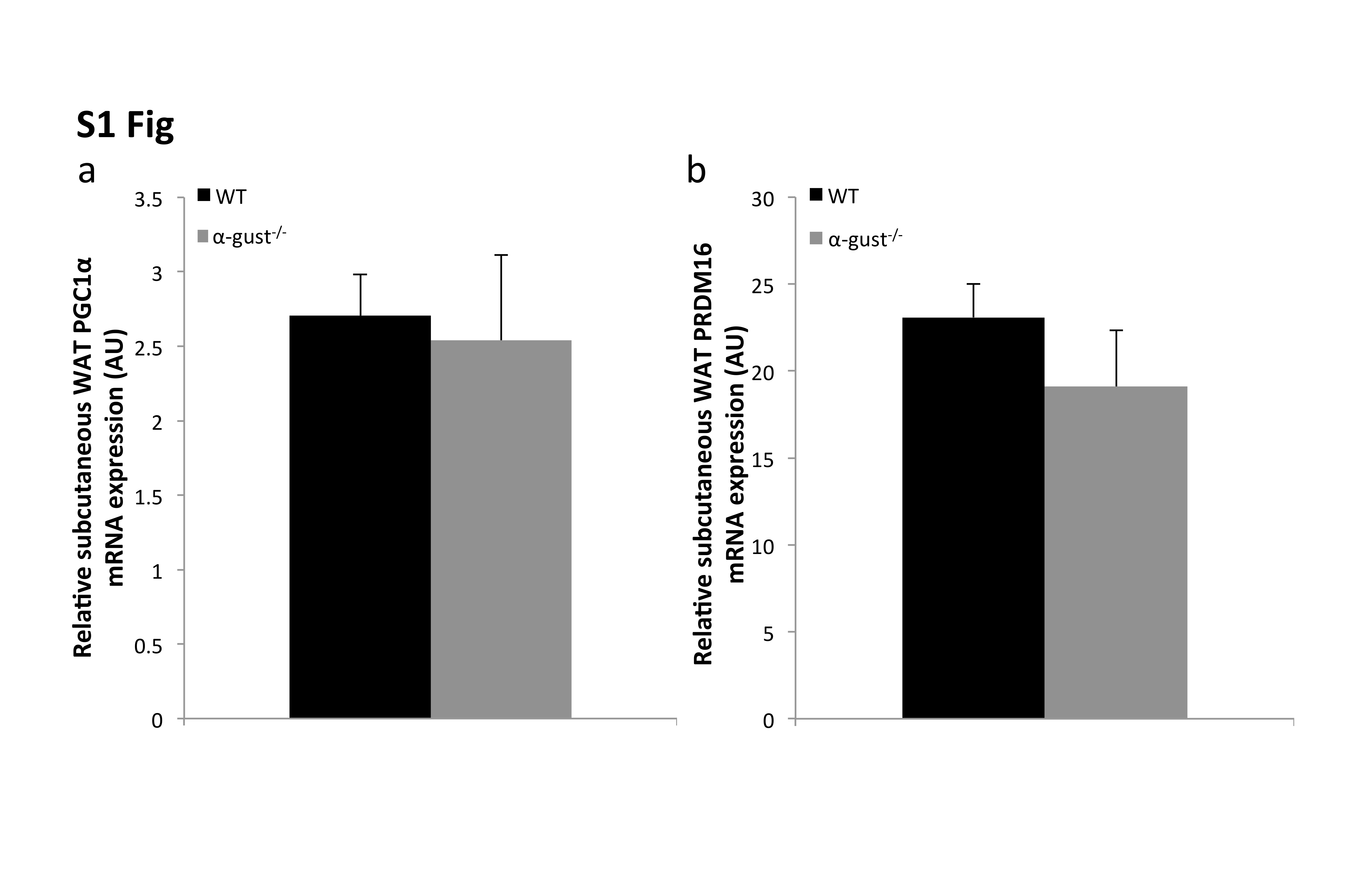

Supplement: S1 Fig — (a) Relative subcutaneous adipose tissue PGC1α (a) and PRDM16 (b) mRNA expression levels in HFD-obese WT (n = 9) and α-gust-/- (n = 9) mice. (TIF) [file pone.0145538.s001.tif]

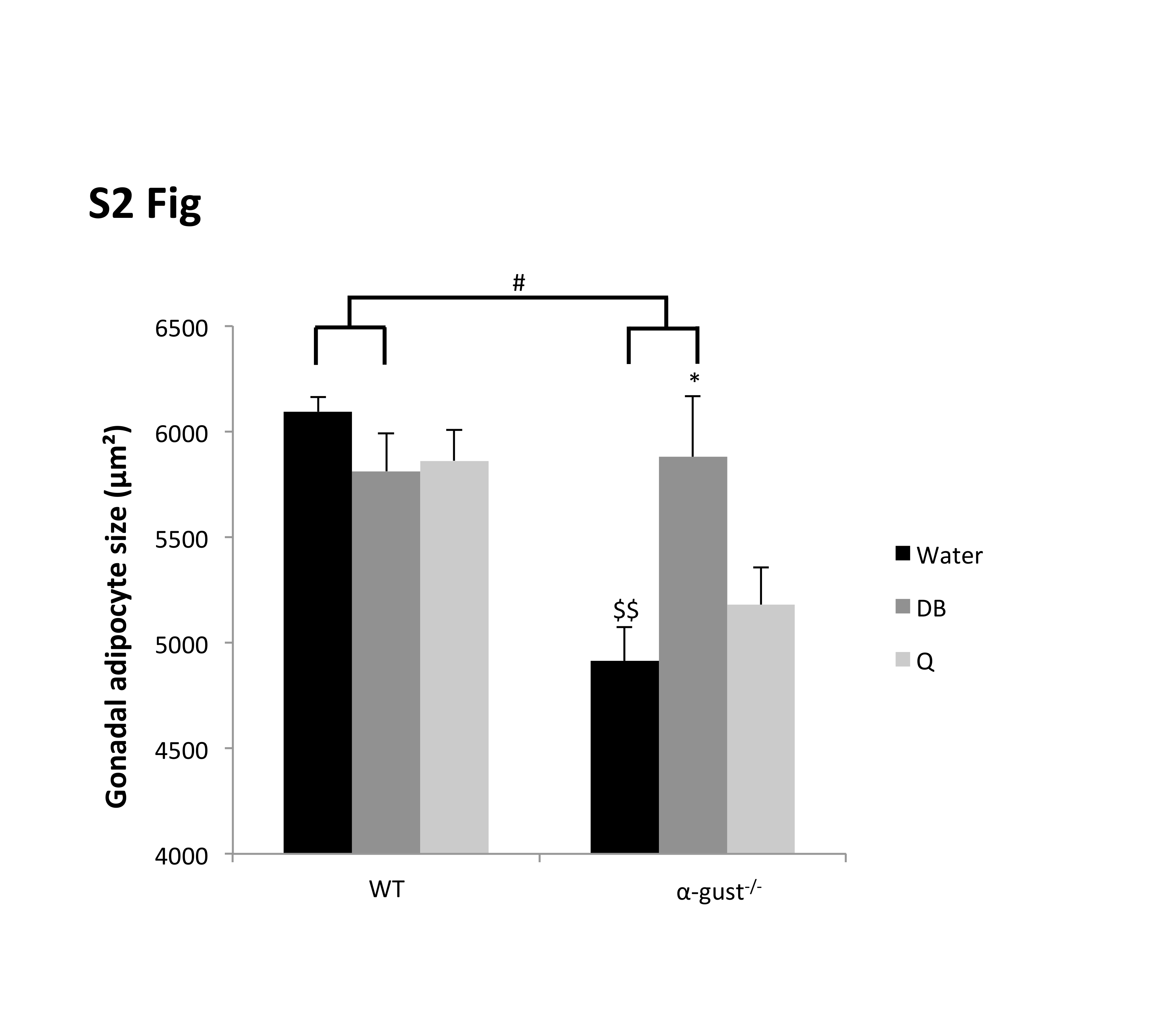

Supplement: S2 Fig — Gonadal adipocyte size in HFD-obese WT (n = 5) and α-gust-/- (n = 6) mice. *: P<0.05 water vs DB; $ $: P<0.01 WT vs α-gust-/-; #: P<0.05 treatment (water vs DB) x genotype. (TIF) [file pone.0145538.s002.tif]

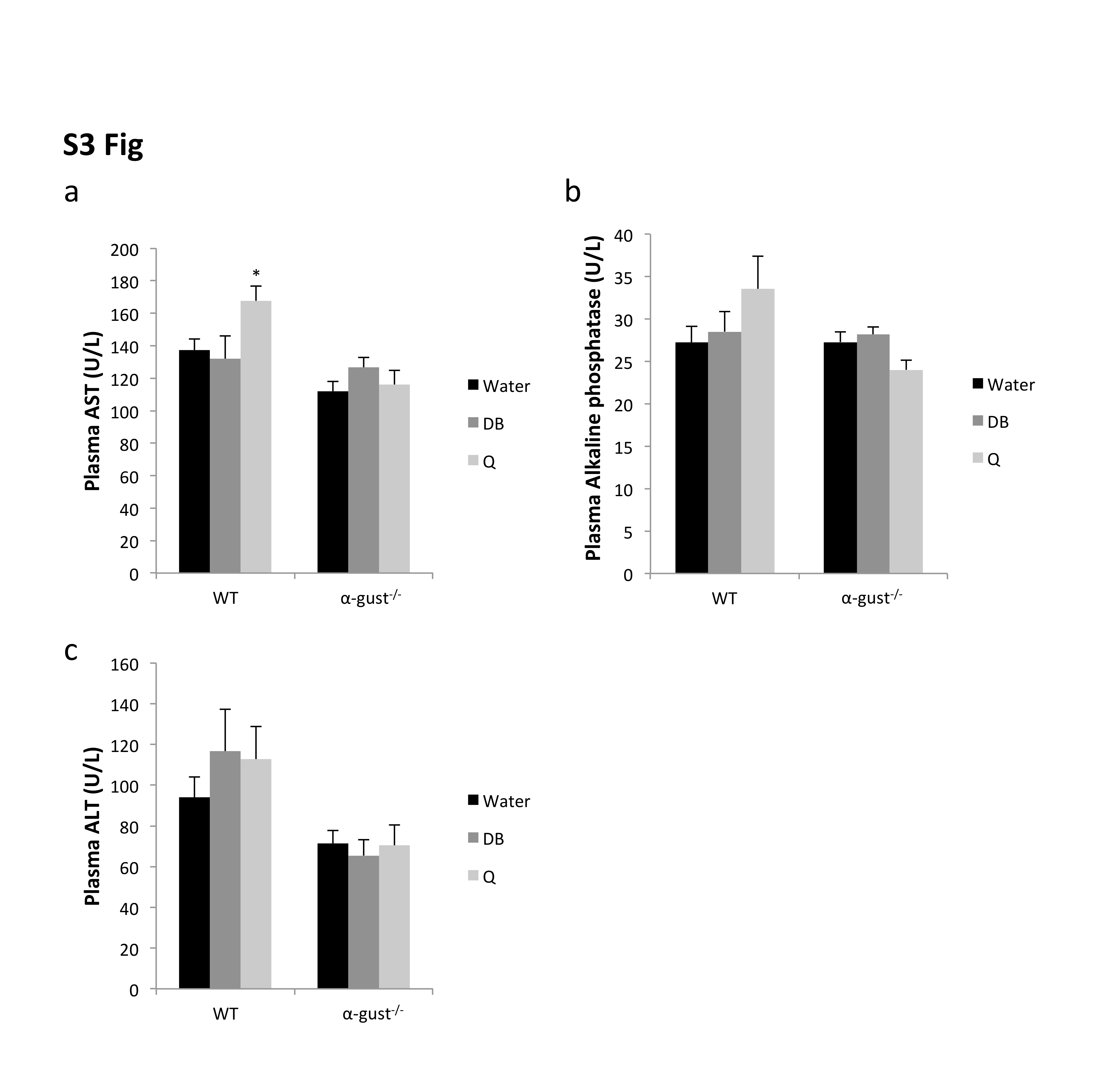

Supplement: S3 Fig — Plasma levels of AST (a), ALT (b) and alkaline phosphatase (c) in HFD-obese WT (n = 8–14) and α-gust-/- (n = 9–13) mice. *: P<0.05 water vs Q. (TIF) [file pone.0145538.s003.tif]

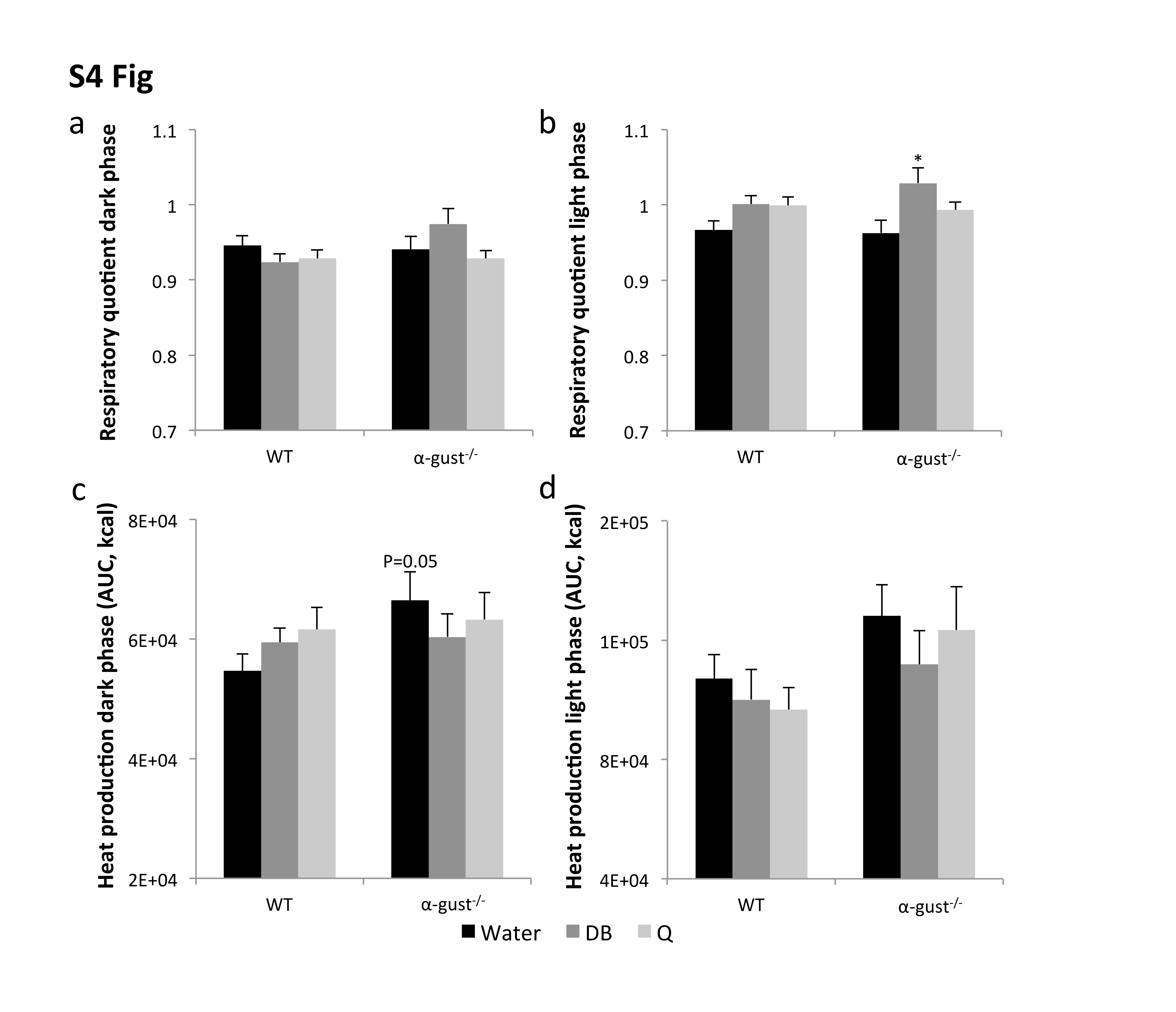

Supplement: S4 Fig — (a-b) Mean respiratory quotient and (c-d) heat production (area under the curve) measured continuously during 1 week in (a, c) the dark and (b,d) the light phase in ad-libitum fed WT (n = 8) and α-gust-/- (n = 8) mice, treated with water, DB or Q for 4 weeks. *: P<0.05 water vs DB. (TIF) [file pone.0145538.s004.tif]

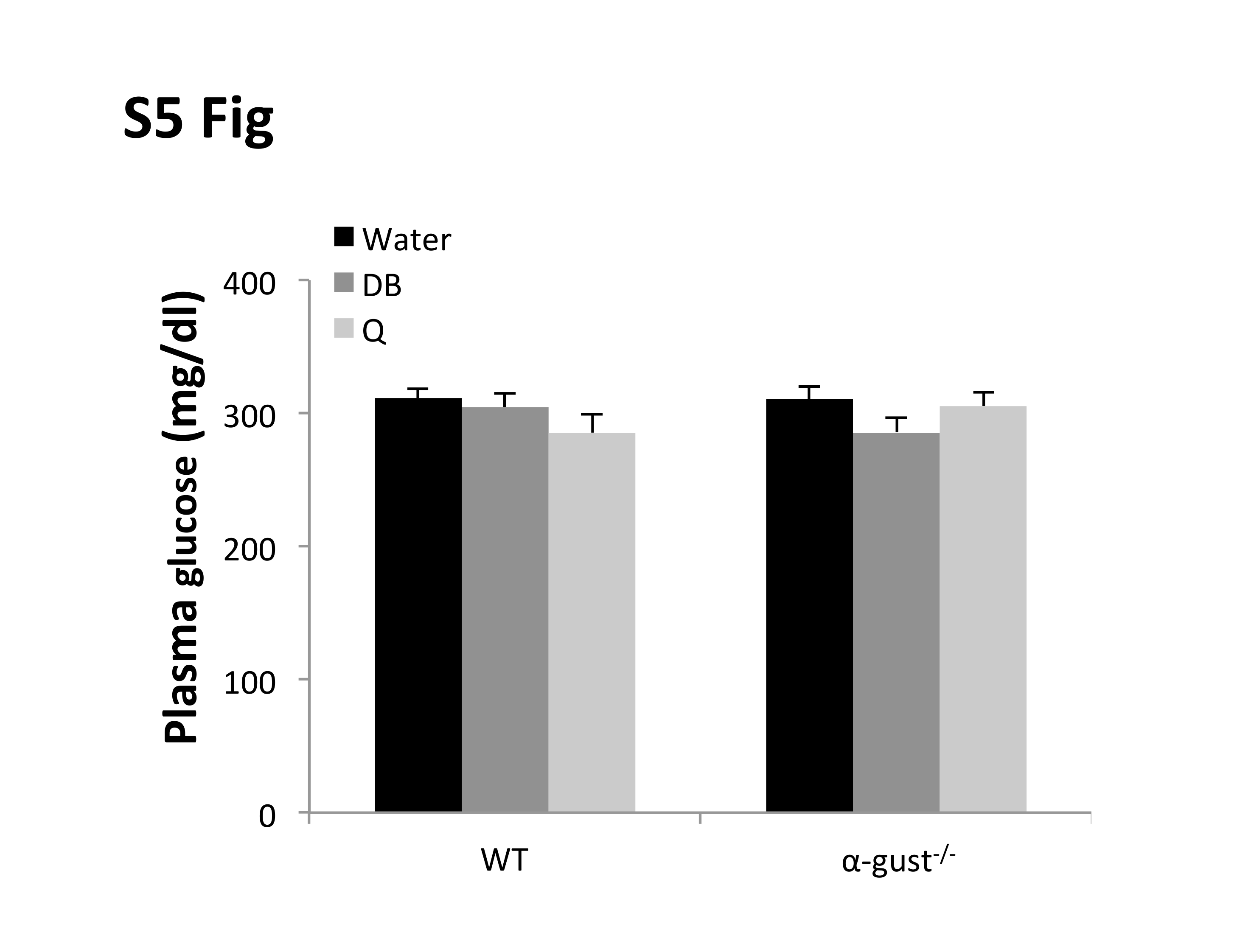

Supplement: S5 Fig — Plasma glucose levels in bitter treated WT (n = 9–12) and α-gust-/- (n = 8–10) mice. (TIF) [file pone.0145538.s005.tif]

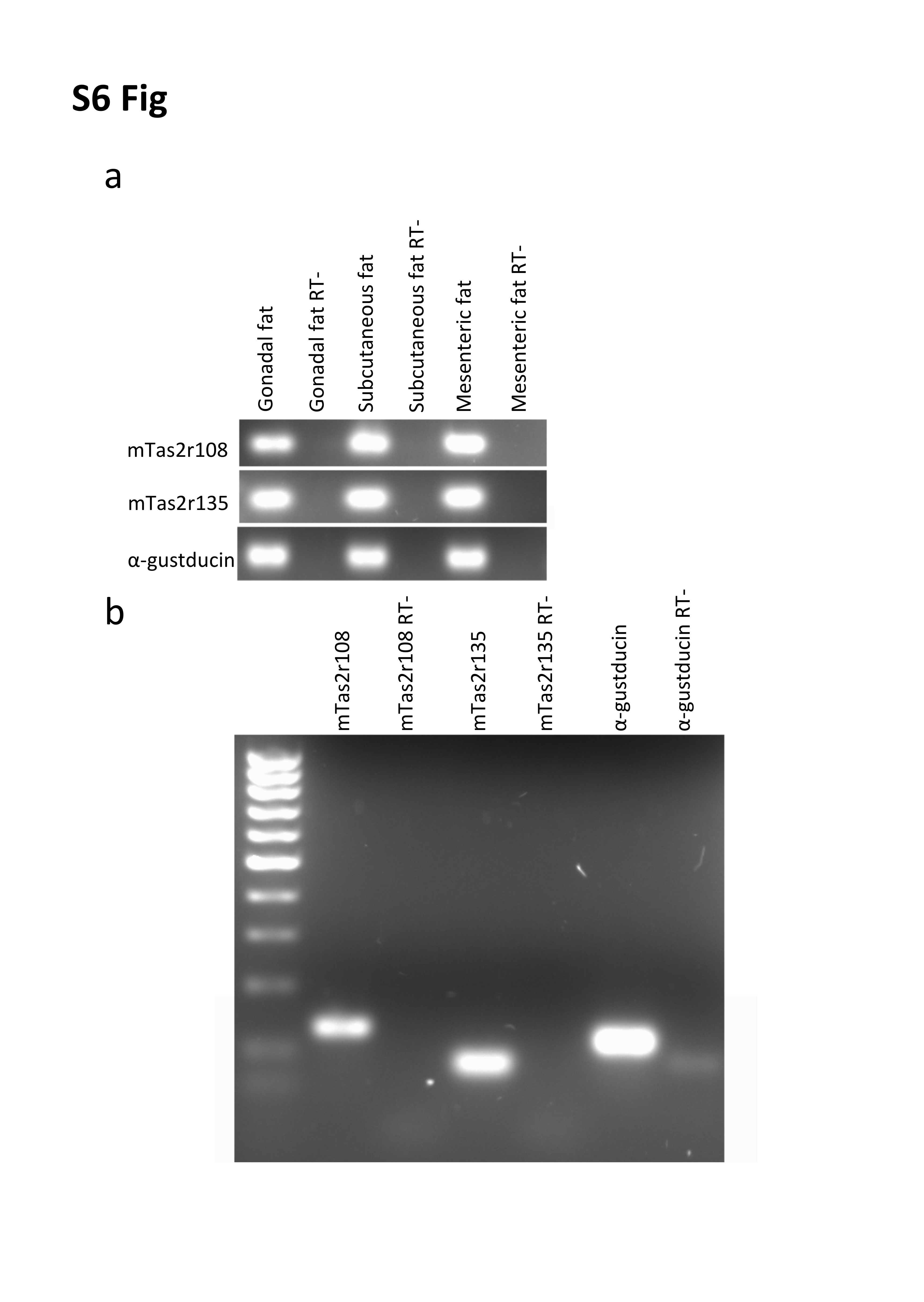

Supplement: S6 Fig — (a) RT-PCR transcripts coding for mTAS2R108 (DB and Q), mTas2R135 (DB) and α-gustducin in obese WT mouse gonadal, subcutaneous and mesenteric fat pads. (b) RT-PCR transcripts coding for mTas2R108, mTas2R135 and α-gustducin in 3T3-F442A cells. Samples in which no reverse transcriptase was added upon production of the cDNA were used as negative control (RT-). (TIF) [file pone.0145538.s006.tif]
