# Supplementary material for: The Gustatory Signaling Pathway and Bitter Taste Receptors Affect the Development of Obesity and Adipocyte Metabolism in Mice
Source: PLoS One. 2015 Dec 21;10(12):e0145538. doi: 10.1371/journal.pone.0145538 (PMC4686985; doi:10.1371/journal.pone.0145538)
Supplement: S1 Table — (DOCX) [file pone.0145538.s007.docx]

S1 Table

| **Gene** | **Forward primer** | **Reverse primer** |
| --- | --- | --- |
| RPL13a | CACTCTggAggAgAAACggAAgg | gCAggCATgAggCAAACAgTC |
| β-Actin | gATCTggCACCACACCTTCTAC | TggATggCTACgTACATggCTg |
| GAPDH | CCCCAATgTgTCCgTCgTg | gCCTgCTTCACCACCTTCT |
| Leptin | CAggATgACACCAAAACCCTC | TCCAAg CAgTgACCCTCTg |
| PPARγ | ACAgACAAgATTTgAAAgAAgCggTgA | TCCgAAgTTggTgggCCAgA |
| AP-2 | CCTTCAAACTgggCgTgg | CgTTTTCTCTTTATTgTggTCgACT |
| FAS | TgggTTCTAgCCAgCAgAgT | ACCACCAgAgACCgTTATgC |
| Adiponectin | gCAgAgATggCACTCCTggA | CCCTTCAgCTCCTgTCATTCC |
| UCP1 | gCCATCTgCATgggATCAA | ggTCgTCCCTTTCCAAAgTg |
| UCP2 | TCACTgTgCCCTTACCATgCT | AggCATgAACCCCTTgTAgAAg |
| Pref-1 | AACCATggCAgTgCATCTg | AGCATTCgTACTggCCTTTC |
| AgRP | gCggAggTgCTAgATCCA | AggACTCgTgCAgCCTTA |
| NPY | CCgCTCTgCgACACTACAT | TgTCTCAgggCTggATCTCT |
| POMC | ACCTCACCACggAgAgCA | gCgAgAggTCgAgTTTgC |
| mTas2R108 | gATTTCAgCCCTCACCACTC | AgTTCAggACCAAAgAggCTAC |
| mTas2R135 | AgCACTgggCATgAAATggT | TCgAgAggCTgCTAgACTgAT |
| α-gustducin | CACCTCCATTgTTCTgTTTCTTAAC | gCATCTTCAAATgTgTTTggTC |
| PRDM16 | CCCTgCCATTgTTAAgACC | TgCTgCTgTTCCTgTTTTC |
| PGD1α | CAgCACggTgAAgCCATTC | gCgTgCATCCgCTTgTg |
