# Supplementary material for: The Gustatory Signaling Pathway and Bitter Taste Receptors Affect the Development of Obesity and Adipocyte Metabolism in Mice
Source: PLoS One. 2015 Dec 21;10(12):e0145538. doi: 10.1371/journal.pone.0145538 (PMC4686985; doi:10.1371/journal.pone.0145538)
Supplement: S2 Table — *: P<0.05 water vs Q; $ $ $ P<0.001 WT vs α-gust-/-. (DOCX) [file pone.0145538.s008.docx]

S2 Table

| **Tissue** | **Weight (mg)** | | | | | |
| --- | --- | --- | --- | --- | --- | --- |
|  | **WT (n=9-12)** | | | **α-gust^-/-^ (n=9-12)** | | |
|  | **Water** | **DB** | **Q** | **Water** | **DB** | **Q** |
| WAT | 6279±187 | 5790±279 | 5544±320^*^ | 4822±249^$$$^ | 5496±387 | 4859±284 |
| BAT | 290±14 | 302±37 | 303±29 | 253±17 | 251±22 | 235±22 |
| Liver | 1994±170 | 2146±238 | 2422±255 | 1995±111 | 1668±130 | 1927±239 |
| Heart | 185±6 | 188±8 | 181±5 | 189±4 | 182±4 | 184±5 |
| Kidneys | 389±17 | 390±10 | 402±9 | 387±13 | 382±8 | 403±13 |
